# Survival of gastrointestinal stromal tumor patients in the imatinib era: life raft group observational registry

**DOI:** 10.1186/1471-2407-12-90

**Published:** 2012-03-19

**Authors:** Jerry Call, Christopher D Walentas, Jens C Eickhoff, Norman Scherzer

**Affiliations:** 1 Life Raft Group, 155 Route 46 West, Suite 202, Wayne, NJ 07470, USA; 2 LPC, 2004 MacGregor Park Circle, Ft Myers, FL 33908-5419, USA; 3 Department of Biostatistics & Medical Informatics, University of Wisconsin, School of Medicine and Public Health, 600 Highland Ave, Madison, WI 53792-4675, USA

## Abstract

**Background:**

Gastrointestinal stromal tumors (GIST), one of the most common mesenchymal tumors of the gastrointestinal tract, prior to routine immunohistochemical staining and the introduction of tyrosine kinase inhibitors, were often mistaken for neoplasms of smooth muscle origin such as leiomyomas, leiomyosarcomas or leiomyoblastomas. Since the advent of imatinib, GIST has been further delineated into adult- (KIT or PDGFRα mutations) and pediatric- (typified by wild-type GIST/succinate dehydrogenase deficiencies) types. Using varying gender ratios at age of diagnosis we sought to elucidate prognostic factors for each sub-type and their impact on overall survival.

**Methods:**

This is a long-term retrospective analysis of a large observational study of an international open cohort of patients from a GIST research and patient advocacy's lifetime registry. Demographic and disease-specific data were voluntarily supplied by its members from May 2000-October 2010; the primary outcome was overall survival. Associations between survival and prognostic factors were evaluated by univariate Cox proportional hazard analyses, with backward selection at *P *< 0.05 used to identify independent factors.

**Results:**

Inflections in gender ratios by age at diagnosis in years delineated two distinct groups: above and below age 35 at diagnosis. Closer analysis confirmed the above 35 age group as previously reported for adult-type GIST, typified by mixed primary tumor sites and gender, KIT or PDGFRα mutations, and shorter survival times. The pediatric group (< age 18 at diagnosis) was also as previously reported with predominantly stomach tumors, females, wild-type GIST or SDH mutations, and extended survival. "Young adults" however formed a third group aged 18-35 at diagnosis, and were a clear mix of these two previously reported distinct sub-types.

**Conclusions:**

Pediatric- and adult-type GIST have been previously characterized in clinical settings and these observations confirm significant prognostic factors for each from a diverse real-world cohort. Additionally, these findings suggest that extra diligence be taken with "young adults" (aged 18-35 at diagnosis) as pediatric-type GIST may present well beyond adolescence, particularly as these distinct sub-types have different causes, and consequently respond differently to treatments.

## Background

### Gastrointestinal stromal tumors

Gastrointestinal stromal tumors (GISTs) are soft tissue sarcomas that originate from the interstitial cells of Cajal (ICC), or from stem cells that can differentiate towards ICCs and can arise anywhere along the gastrointestinal tract (GI), and elsewhere in the abdomen with a reported incidence of up to 14.5 per million per year [1]. Primary tumors most commonly occur in the stomach or small intestine, and most frequently metastasize to the liver or peritoneum [2]. The reported median age of GIST patients varies from 54 to 67 years [3-6].

KIT and PDGFRα genes code for their respective tyrosine kinase receptors, and known mutations result in constitutive activation driving the proliferation and survival of GIST tumor cells. KIT mutations occur in about 75-80% of GISTs and about 7% have PDGFRα gene mutations [5]. KIT and PDGFRα mutations are mutually exclusive. About 15% of GISTs do not have mutations in either KIT or PDGFRα and are commonly referred to as wild-type GIST.

Cytotoxic chemotherapy is ineffective in GIST, and prior to the introduction of tyrosine kinase inhibitors, the prognosis for patients with metastatic disease was poor with a median OS < 2 years [7]. The prognosis for GIST changed dramatically after the introduction of effective targeted treatments beginning with clinical trials for imatinib in 2000, and its subsequent approval for advanced GIST in 2002; sunitinib was approved for imatinib refractory/intolerant GIST in 2006; approval for adjuvant imatinib in 2008 (EU, 2009). Imatinib is an oral, small-molecule tyrosine kinase inhibitor initially developed as a BCL-ABL inhibitor for chronic myelogenous leukemia. Imatinib also inhibits KIT and PDGFRα, and has proved to be highly effective for GIST, with approximately 85% of patients receiving significant clinical benefit [3,4,6].

KIT mutations in GIST occur throughout the gene. The most common encodes the juxtamembrane domain, exon 11 (60-70% of all GISTs), followed by the extracellular domain, exon 9 (10-15%), the kinase I domain, exon 13 (2%), and activation loop, exon 17 (1.3%) [3,4,8]. The mutational status of GISTs has proven to be highly predictive of response to modern therapies. For first-line therapy, exon 11 mutants respond well to imatinib, but KIT exon 9 mutations respond less favorably, and appear to require a higher dose of imatinib [8]. While there is much less clinical data, most of the more rare mutations, KIT exons 13 and 17, and PDGFRα exons 12 and 14, are sensitive to imatinib *in vitro *[9]. The most common PDGFRα mutation (62.6% of PDGFRα mutations [5]), the exon 18 D842V mutation, is insensitive to imatinib [9].

### GIST survival

Reported survival times for GIST patients have varied widely depending on the era (pre-imatinib, imatinib era or during the transition), selection criteria, and the starting point used for measuring survival. Several early reports from GIST referral centers suggested that 90% or more of GISTs (thought to be gastrointestinal leiomyosarcomas at the time), would eventually have a recurrence, and that most patients would eventually die as a result [10,11]. For example, Nu and colleagues reported in 1992 a 5-year survival rate of 28% and a median survival of 29 months [10]. These early reports probably were influenced by referral bias and represented primarily advanced/metastatic disease.

Survival for advanced/metastatic GIST in the imatinib era has been reported for several trials. A phase II trial of 147 GIST patients reported a median OS of 57 months (B2222 [12]), a phase III trial of 746 US/Canadian patients had a median OS of 53 months (S0033 [13]), and a larger phase III trial of 946 European/Australasian patients described a median OS of 45 months (62005, [13]).

Overall survival data for GIST patients taking adjuvant imatinib is limited. In a randomized trial comparing 12 months of adjuvant imatinib versus 36 months of adjuvant imatinib, patients assigned to 36-months of imatinib had longer OS (HR = 0.45, 95% CI = 0.22-0.89; P = 0.019) [14]. With a median follow-up of 54 months, 92% of patients receiving 36 months of adjuvant imatinib were alive compared to 82% alive for patients receiving 12 months of imatinib. All of these survival times were calculated from the date of trial entry until death. Other studies may calculate overall survival from the date of diagnosis, which can often precede enrollment, potentially leading to prolonged observed survival compared to other results.

Beginning in 2002, reasonably effective criteria for estimating the risk of recurrence have been developed [15-18]. Using newer population-based studies, larger institutional studies, and emerging data from adjuvant imatinib trials, it is now clear that a significant portion of GISTs have a low to very low risk of recurrence. For example, preliminary data from the Z9001 adjuvant imatinib trial reported that 45% of patients enrolled were at low risk according to the Miettinen criteria of recurrence [19].

### Pediatric and adult GIST

GIST in children is rare and patients under the age 18 account for 1-2% of cases. In addition to GIST, pediatric patients often develop paragangliomas and/or chondromas. When any two of these three are present, it supports a diagnosis of Carney's Triad [20].

The etiologies of adult and pediatric GIST appear to be different, with adult GISTs being primarily driven by KIT or PDGFRα mutations. In 2007, Pasini *et al. *reported in some familial GIST patients, germline mutations in the succinate dehydrogenase subunits SDHB, SDHC, and SDHD [21]; SDH mutations had been previously reported in patients with familial paraganglioma [22]. SDH subunit A mutations have also recently been implicated in GIST [Pantaleo MA, JNCI 2011; Pantaleo MA, Am J Surg Pathol 2011][23]. In a series of pediatric and wild-type patients seen at the National Institute of Health pediatric & wild-type GIST clinic, 4 of 34 (12%) patients had germline SDH mutations [24]. Other groups have since reported a loss of SDHB expression in pediatric GIST patients even when there was no known mutation [25,26]. Taken together, these findings suggest that loss of function of SDH, a tumor suppressor, can be a causative factor in pediatric GIST.

There are considerable differences between pediatric GIST and those that occur in adults. Up to 85% of pediatric GIST patients are female [27], whereas a little under half that number of adult patients are female (45%) [3,4,6]. Pediatric primary tumors tend to be multi-focal, with epithelioid histology and occur predominantly in the stomach. Adult GIST primary tumors however, can originate anywhere along the GI tract and tend to present singly, with spindle cell histology. Metastases are common in both types, however, metastases to the lymph nodes are common in pediatric GIST, but rare in adult GIST [28]. In spite of limited effectiveness of targeted therapies for pediatric GIST, extended survival times have been reported [29,30].

## Methods

### Study design

This report describes a retrospective analysis of a long-term observational study of overall survival in a large open-cohort of patients diagnosed with GIST. Subjects represent the lifetime membership registry of the Life Raft Group (LRG), an international, internet-based private, non-profit (501.c.3) medical research and patient advocacy organization established to champion GIST patients and facilitate related research.

### Setting and subjects

The LRG registry contains data provided by its members worldwide, recruited by referrals from attending physicians or other GIST patients. The majority of members however were acquired through patient-initiated contacts, following internet searches or other requests for disease-related information and assistance. All members had a reported initial diagnosis of GIST. There were no other criteria for membership, and no one was excluded owing to age, location, confirmation of diagnosis, disease status, or prior or current treatment regimen or response.

Acquisition of data from members was strictly on a voluntary basis. All data were collected either using electronic questionnaires periodically forwarded as reminders to members, a relative, or caregiver, or through phone interviews. Demographic data included gender, date and country of birth and country of treatment. Disease-specific data included date and disease status at diagnosis, disease status at last update, primary tumor location, and tumor mutation data as available. Deaths were most often reported by family members, many of whom are also Life Raft Group members. In addition, public death records, such as the social security death index (SSDI) were reviewed for members without a recent update.

In addition to disease status at initial diagnosis *e.g.*, metastatic/not metastatic, the confidence with which a report of "not metastatic" was estimated and coded into the database *post hoc*. Those who recently and regularly provided updates were deemed of "high confidence". "Low confidence" was attributed to those who rarely, if ever, provided an update beyond initial registration. The remainder, those who had provided periodic, but not necessarily a recent update (within ~6 months prior to cohort analysis), were considered of "intermediate confidence". Only those deemed of high confidence are included in the survival analysis of non-metastatic patients. Questionnaire responses were reviewed, and the data were coded, logged, and curated by LRG staff, who also confirmed all end points, *e.g.*, death event; the primary outcome measure for this study was overall survival.

This study was conducted under guidelines consistent with US and international policies regarding research involving human subjects. All subjects were informed as to the nature and goals of this study. Subjects had the right to withdraw participation at any time for any reason. While voluntarily obtained, all data were nonetheless collected and maintained in adherence to regulations on data involving human subjects.

### Statistical methods

Categorical outcomes were analyzed descriptively and summarized in terms of frequency tables. Means and standard deviations were used to summarize all variables measured on a quantitative scale. The associations between overall survival and gender, age, primary tumor location, presence of metastatic or advanced disease at diagnosis, and mutation status were evaluated by performing univariate Cox proportional hazard analysis. Backward selection with a *P*-value cut-off of < 0.05 was used to identify independent prognostic factors for overall survival. The proportional hazard assumption was verified using plots of the log(-log) survival curves and Schoenfeld residual plots.

All statistical tests were two sided, and *P*-values < 0.05 were considered significant. Statistical data analyses were performed using SAS statistical software (version 9.2, SAS Institute Inc., Cary, NC). Kaplan-Meier curves were generated using GraphPad Prism version 5.04 for Windows, GraphPad Software, San Diego California USA, http://www.graphpad.com. All other calculations were performed using Microsoft Office Excel 2003.

## Results

### Patient- and disease-related characteristics

Member data were collected from May, 2000 to October, 2010. As of October 12, 2010, the LRG GIST registry contained 1233 members. Eighteen members (7 males), were excluded from the final analysis (4 GIST misdiagnoses, 14 actively opted out). The final cohort included 1215 patients (51.7% male), with a median age at time of diagnosis of 52 years (range, 5-92); 312 events (62.5% male) were recorded. Patients were reported to be from 75 different nations and treated in 48 different countries, however the majority of patients were either from (75.7%), or treated in (70.0%), the US. Country of origin and treatment were grouped *post hoc *by continent or as unknown (Table 1).

**Table 1:** Patient data by gender

	Females	Males	All patients
Total cohort (N)	587	628	1215
Missing date of birth (n)	9	12	21
Missing date of diagnosis (n)	5	7	12
Median age at diagnosis in years [range]	50.5 [5.4-92.2]	52.6 [10.0-90.0]	51.7 [5.4-92.2]
Median follow-up time in years [range]	4.5 [0-39.5]	5.8 [0-30.6]	5.2 [0-39.5]
Death events (n)	117	195	312
Total survival cohort (n)	578	617	1195
Primary tumor location (n)	523	565	1088
Tumor site, survival (n)	519	556	1075
Disease status at diagnosis (n)	587	628	1215
Disease status, survival analyses (n)	578	617	1195
Confidence in no metastatic/advanced disease report (see methods for description)			
High confidence	114	100	214
Intermediate confidence	102	96	198
Low confidence	5	12	17
Report of metastatic/advanced disease	366	420	786
Mutation data (n)	164	161	325
Mutation, survival (n)	164	160	324
Continent of birth (n)			
N. America	468	489	957
Europe	69	71	140
Asia	30	43	73
Oceania	7	9	16
S. America	6	10	16
Africa	6	3	9
Unknown	1	3	4

Patients missing a date of birth were not included in analyses based on age at diagnosis (n = 21). Follow-up was calculated from date of diagnosis until date of last update (or endpoint) in years. Survival cohort and sub-groups includes those patients for which overall survival could be estimated. Continents of birth were grouped *post hoc *from reported country of birth

One quarter of all patients (23.5%, 285/1215) reported advanced or metastatic disease at diagnosis, increasing to nearly two-thirds (63.2%, 768/1215) by last update. Those not reporting advanced or metastatic disease at last update were divided into three groups by confidence in these data: high (n = 209), intermediate (n = 188), or low (n = 15). Overall survival by disease status at diagnosis could be calculated for 1195 patients.

Primary tumor site location was provided as free text and grouped *post hoc *as either stomach, small intestine, rectum/anus, esophagus, multiple gastrointestinal (mult. GI), other, or unknown. Nearly all cases in the < 18 at diagnosis age group (26/28), and all tumors in females aged < 18 years at diagnosis (21/21) were reported in the stomach; two males in this youngest age group reported primary tumors in the small intestine. Gender differences in primary tumor location became less evident with increasing age, and older age groups reported a greater range of primary tumor sites (Table 2).

**Table 2:** Reported primary tumor location by age group and gender

	< 18		18-35		> 35		Total
	
Primary Tumor Location	F	M	F	M	F	M	
Stomach	21	5	47	18	198	210	499
Small intestine		2	25	12	148	204	391
Unknown			4	6	54	50	114
Colon				1	16	16	33
Rectum/anus				2	13	17	32
Large & small intestine				2	5	9	16
Stomach & small intestine					6	5	11
Esophagus & stomach			2		3	6	11
Omentum					4	7	11
Esophagus			1		3	6	10
Peritoneum			1		5	4	10
Other				1	5	3	9
Mesentery			1		2	5	8
Pelvis			1		2	5	8
Liver				1	3	2	6
Retroperitoneal				1		4	5
Pancreas					2	2	4
Spleen					1	2	3
Chest						1	1
Adrenal gland					1		1
Bladder					1		1

Total	21	7	82	44	472	558	1184

Primary tumor sites were provided as free text as listed below in decreasing order. Care must be taken in the interpretation of these data as reporting of primary tumor location as a single or unique site can be challenging (see Limitations). Patients of unknown age at diagnosis were not included (n = 31)

### Age at diagnosis by gender

**Figure 1:**
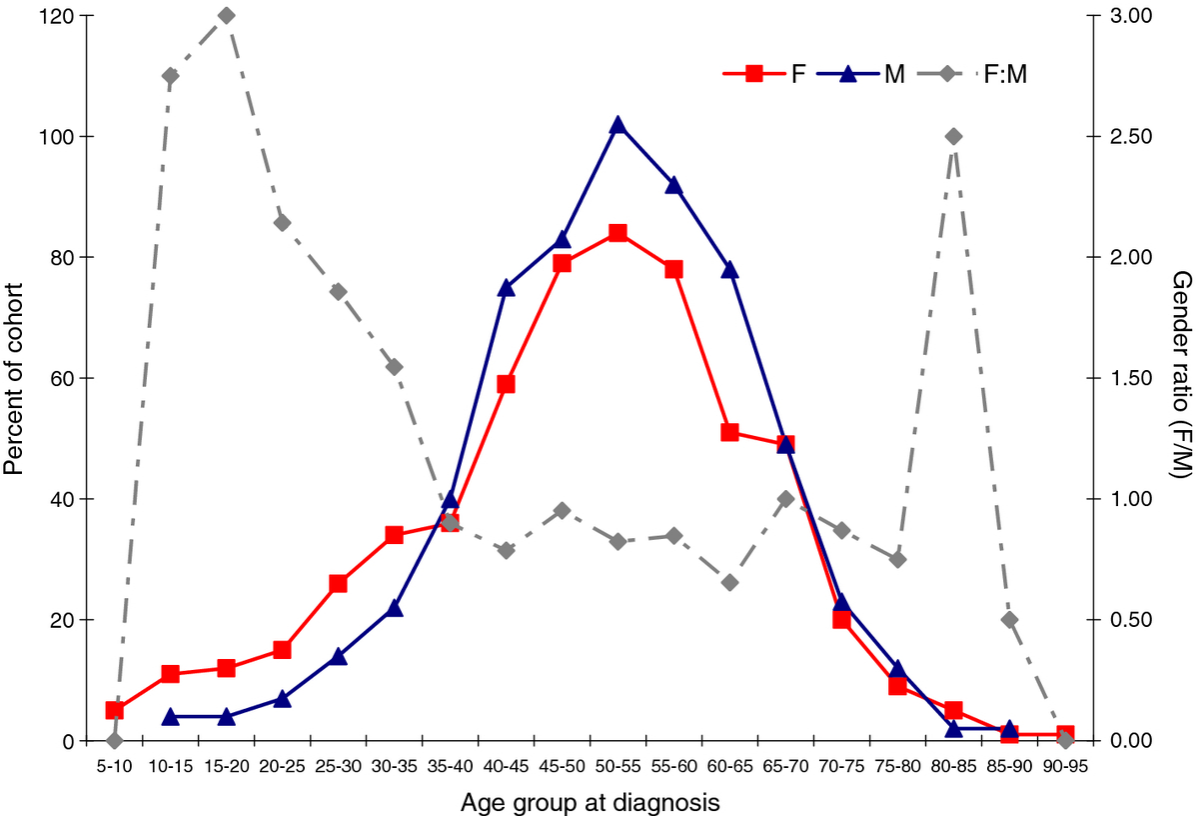
**Age at diagnosis by gender**. Female gender is known to be associated with pediatric-type GIST. Using high female to male ratio as a surrogate marker for pediatric GIST, our data suggests that pediatric-type GIST occurs with decreasing frequency until about age 35 (at diagnosis). Also see Table 3.

A distribution of patients by age at diagnosis and by gender revealed two distinct populations (Figure 1, Table 3). Eight-seven percent of the cohort was diagnosed > 35 years old (54.2% male), 2.4% were diagnosed before the age of 18 (25.0% male), the remaining "young adult" sub-group (10.6%, diagnosed at 18-35 years) displayed a gender balance between these other two groups (34.9% male). There were some patients (n = 31, 2.6%) for which an age at diagnosis could not be determined (21 missing date of birth, 12 missing date of diagnosis).

**Table 3:** Gender ratio by age at diagnosis

Age group at diagnosis	Female	Male	Gender ratio (F:M)
5-10	5		-
10-15	11	4	2.75
15-20	12	4	3.00
20-25	15	7	2.14
25-30	26	14	1.86
30-35	34	22	1.55
35-40	36	40	0.90
40-45	59	75	0.79
45-50	79	83	0.95
50-55	84	102	0.82
55-60	78	92	0.85
60-65	51	78	0.65
65-70	49	49	1.00
70-75	20	23	0.87
75-80	9	12	0.75
80-85	5	2	2.50
85-90	1	2	0.50
90-95	1		-

N/A	12	19	0.63

### Mutational analysis

Three hundred twenty four patients (26.7%) reported tumor mutation data. Half (53.6%, 15/28) of patients aged < 18 and a quarter (25.5%, 261/1022) of those > 35 years reported mutation data, with a "young adult" population (18-35 years at diagnosis) reporting at an intermediate rate (38.1%, 48/126). Wild-type (wt) mutations were reported in 10 pediatric female patients, with 2 reports of a mutation within SDH subunit C; there were 2 wild-type GISTS and 1 KIT exon 11 mutation (familial) reported in males of this youngest age group (Table 4). The "young adult" group contained 5-fold more females than the pediatric group and wild-type GIST and SDH mutations comprised 56% of this middle group's (18-35) mutational reports. However, when gender was considered, wild-type GIST was reported almost exclusively by females, with a single report of an SDH mutation in a male in this age group. The adult population contained a closer balance of genders and mutations, as well as a greater variety of reported mutations with 8% reporting wild-type GIST (12 females, 8 males) or SDH mutations (1 female).

**Table 4:** Mutation by age group and gender

Gene	Exon/subunit	< 18		18-35		> 35		Total
		**F**	**M**	**F**	**M**	**F**	**M**	

KIT	11		1	8	6	71	100	186
	9			3	3	18	20	44
	13					2	5	7

Wild-type		10	2	25		12	8	57

PDGFRα	18				1	7	10	18
	12					3	2	5
	17					1		1
	Unknown						1	1

SDH	C	2						2
	B			1	1			2
	Unknown					1		1

Unknown		9	4	45	33	352	409	852

Grand Total		21	7	82	44	467	555	1176

Mutational status was reported by nearly one third of the cohort (n = 325). Patients of unknown age at diagnosis were not included (n = 31). Pediatric-type mutations include the wild-type GIST and SDH mutations. Wild-type GIST is a characteristic of pediatric-type GISTs, and 12 of 15 patients aged < 18 at diagnosis reported wild-type GIST. However, wild-type GIST is a heterogeneous mix that includes both adults and pediatric patients. KIT and PDGFRα mutations are broken out by exon, where known, whereas mutations within the succinate dehydrogenase complex denote subunits

### Overall survival by gender, age at diagnosis and reported disease status, tumor site and mutation

**Figure 2:**
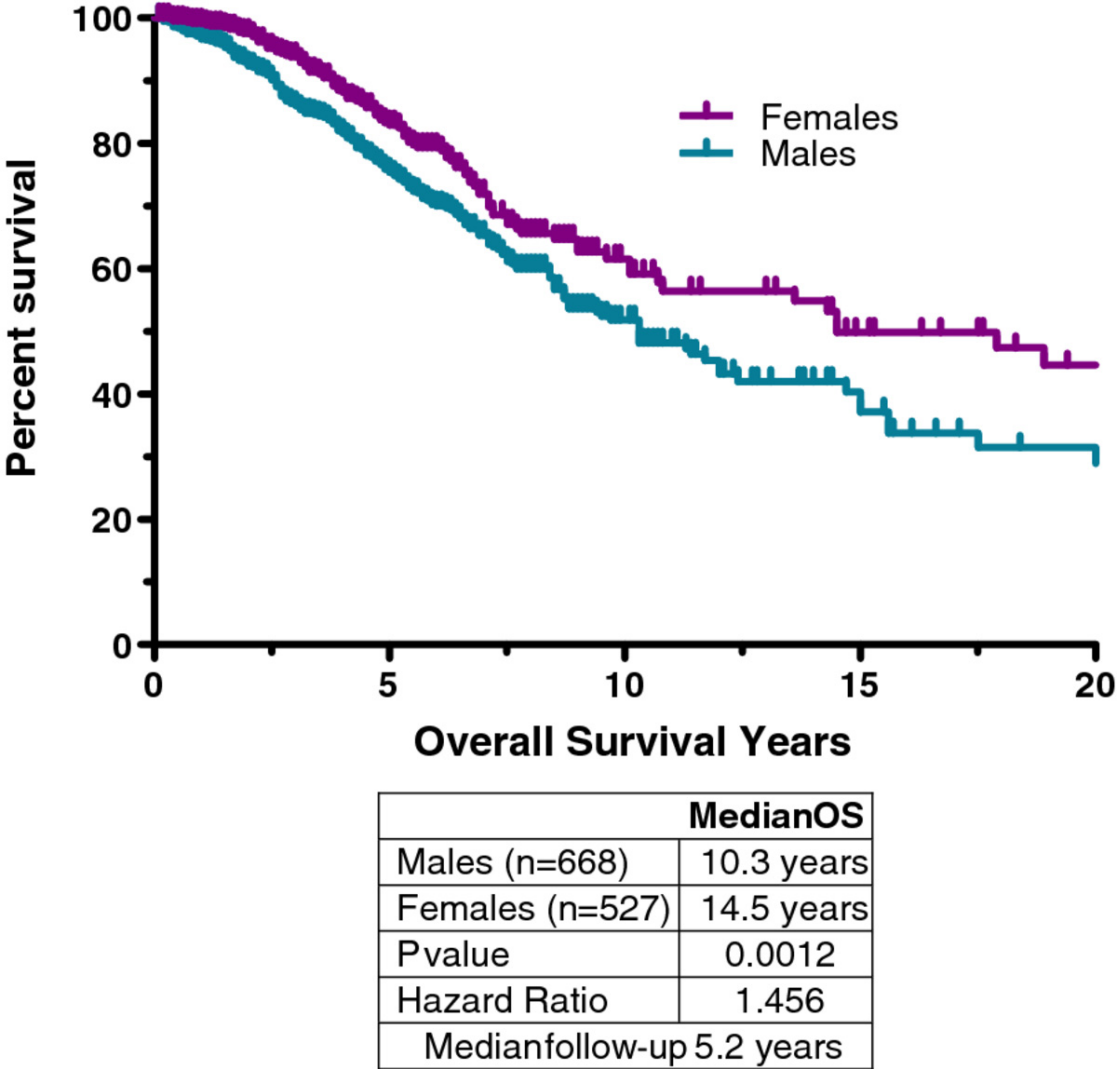
**Overall survival: males vs. females**. Females displayed significantly longer overall survival than males. This appears to be influenced by the long survival times and greater number of females in those with pediatric-type GIST. A smaller, but significant survival advantage was still noted for females > 35 at diagnosis (see Discussion)

Overall survival (OS) could be calculated for 1195 patients (51.6% male) for which there were a total of 312 recorded deaths (62.5% male). The median OS for the entire cohort was observed to be 11.7 years, with a 79% 5-year OS. When gender was considered, females showed a 4.2 year median OS benefit over males (P = 0.0012, HR = 1.456, 95% CI = 1.2-1.8; Figure 2) with a median OS of 14.5 years for females and 10.3 years for males.

**Figure 3:**
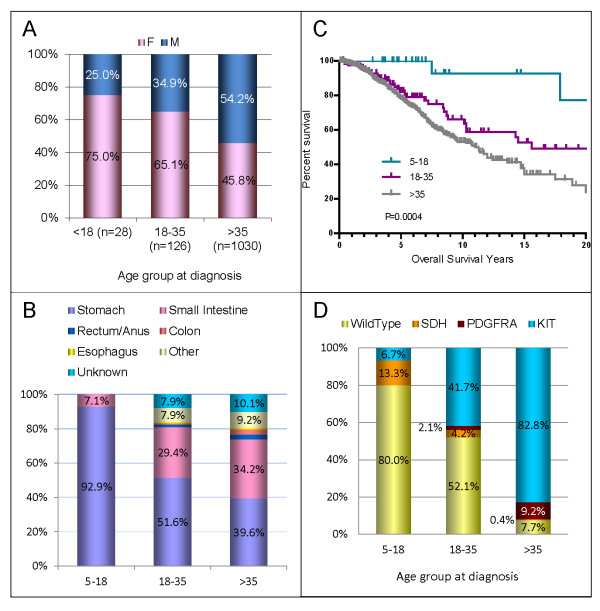
**Patient and disease characteristics by age group at diagnosis**. Patients diagnosed below the age of 18 had characteristics associated with pediatric-type GIST: female gender (panel A), predominantly stomach primaries (panel B), prolonged overall survival (panel C), and wild-type GISTor SDH mutations (panel D). Patients diagnosed over age 35 had characteristics associated with adult-type GIST: slight male preference (panel A), slight preference for stomach primary tumor location followed closely by small intestine primaries (panel B), shorter overall survival (panel C), and dominated by KIT mutations (panel D). The 18-35 at diagnosis age group had characteristics between those of pediatric-type and adult GIST. Tumor locations were provided as free text and are depicted as a relative percentage within each age-group (panel B). Some sites have been grouped *post hoc *including, multiple GI (stomach and esophagus, stomach and small intestine, small and large intestine), and "other" which includes all remaining reported primary tumor sites (table 2); the rest were as reported (esophagus, stomach, small intestine, colon or rectum/anus). This was an attempt to adhere to published groupings without changing the data as reported. Multi GI, colon, and rectum/anus in the > 35 group, and multi GI in the 18-35 group were ~3% (2.9-3.3%); the remaining unlabelled sites were all < 2% of age-group (0.8-1.6%).

When placed into age groups as previously described for age at diagnosis, the median OS for patients > 35 was 11.3 years, and 15.6 years for those diagnosed 18-35; the median OS for patients diagnosed < 18 years had not been reached at time of analysis (P = 0.0004; Figure 3C).

**Figure 4:**
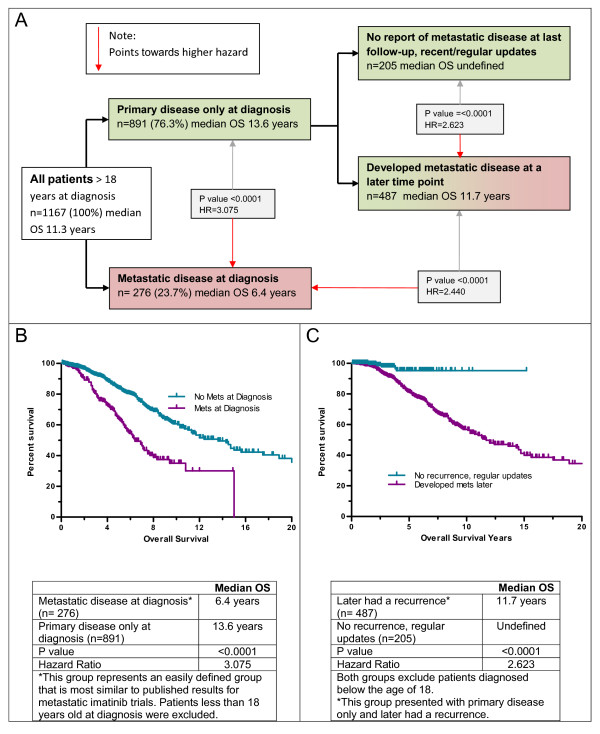
**Overall survival by disease stage**. The effect of disease stage on overall survival for patients > 18 years at diagnosis is shown in panel A. Patients that had metastatic disease at the time of diagnosis did significantly worse than patients that presented with primary disease only (panel B). Patients that had a recurrence later still did relatively well, but predictably, not as well as patients without a recurrence (panel C).

Disease status at time of diagnosis also had a significant impact upon survival (Figure 4A), with a 6.9 year median OS observed for patients reporting metastatic or advanced disease at diagnosis compared to 14.5 years for those without metastatic/advanced disease at diagnosis (P < 0.0001, HR = 2.9, 95% CI = 2.2-3.9). The closest approximation of the registry cohort to the population of published metastatic trials would exclude all pediatric patients (< 18 years at diagnosis). When this post-adolescent subgroup was analyzed for survival by report of advanced/metastatic disease at diagnosis, the median OS for the all-adult "no mets at diagnosis" group was 13.6 years, however the median OS for those reporting advanced/metastatic disease at diagnosis was 6.4 years, ~7 fewer years (Figure 4B). Patients > 18 at diagnosis, and presenting with primary disease only, were divided into two groups: those who later developed a recurrence and those who provided recent and regular updates and never reported a recurrence (median OS was 11.7 years and undefined respectively, P = < 0.0001, HR = 2.62, 95% CI = 1.7-4.1, Figure 4C). Patients reporting advanced or metastatic disease at any time displayed a median OS of 10.3 years.

**Figure 5:**
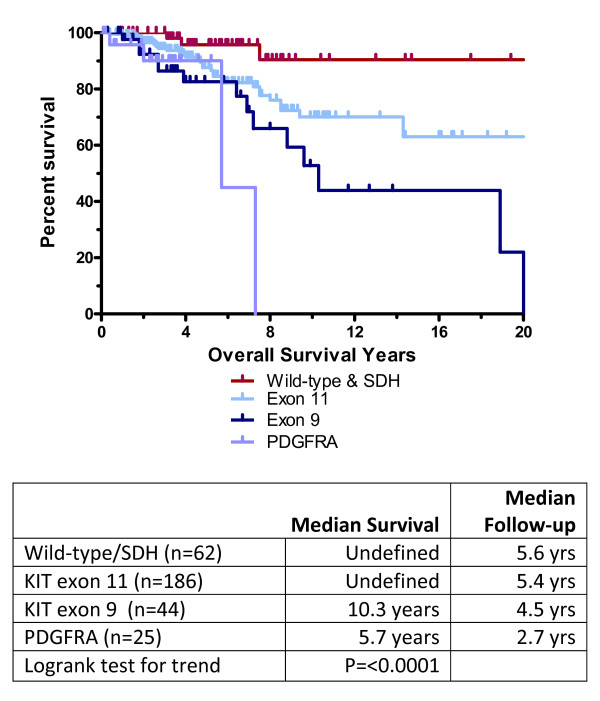
**Overall Survival by Genotype**. When calculated from the time of diagnosis, patients with wild-type GIST/SDH deficiencies or KIT exon 11 mutations did better than other types. The long survival times for wild-type GIST/SDH deficiencies in this series may reflect a high percentage of pediatric-type GIST patients. The relatively short survival time noted for PDGFRα mutations should be interpreted with caution given the small numbers and short follow-up.

In contrast to other reports, when wild-type GIST and SDH patients were combined, their median OS appeared to be better than other types including those with exon 11 mutations (Figure 5), however both wild-type/SDH and exon 11 median OS remained undefined. Patients with exon 9 mutations had a median OS of 10.3 years and those with PDGFRα mutations had a median OS of 5.7 years, but care should be used in interpreting the PDGFRα data since only 4 events occurred and median follow-up time was short (2.7 years) (logrank test for trend, P ≤ 0.0001, Figure 5).

### Factors in overall survival

A Cox-proportional hazards regression model was used to evaluate the association between reported patient and disease characteristics and observed overall survival as calculated from the date of diagnosis. Gender, age at diagnosis, primary tumor location and report of advanced or metastatic disease at diagnosis were identified as independent significant factors. Male gender was a negative predictor of survival (male vs. female, HR = 1.5, P = 0.0010). Older age at diagnosis was also a significant predictor of OS when compared to pediatric patients (> 35 vs. < 18, HR = 8.4, P = 0.0031; 18-35 vs. < 18, HR = 5.8, P = 0.0167); survival in the "young adult" age group approached significance compared to the oldest age-group at diagnosis (> 35 vs. 18-35, HR = 1.4, P = 0.0782). The report of advanced or metastatic disease at diagnosis was also significant (present vs. absent, HR = 2.4, P < 0.0001), however while primary tumor location as a factor was significantly associated with OS, only small intestinal tumors were found to be significant (small intestine vs. other HR = 0.7, P = 0.0390).

## Discussion

Since the introduction of imatinib in clinical trials in 2000, and its subsequent FDA approval for KIT-positive GIST in Feb. 2002, survival of patients with advanced/metastatic GIST has been well characterized within controlled clinical settings. Survival of patients without advanced disease, or otherwise ineligible for trial enrollment has been less well characterized. This observational registry reports on the survival of GIST patients at a range of ages, locations, and disease statuses. Many of these patients participated in clinical trials, but most did not.

GISTs have been previously divided into two distinct sub-groups: adult, primarily displaying KIT or PDGFRα mutations [31,32] and pediatric GIST typified by wild-type GIST or deficiencies in the succinate dehydrogenase complex [26,30,33]. In our cohort of patients, 2.4% were diagnosed before the age of 18, 10.6% were diagnosed between the ages of 18-35, and 87.0% were diagnosed after the age of 35. The youngest group was dominated by characteristics previously ascribed to pediatric-type GIST: female (75%; Figure 3A), stomach primary tumors (93%; Figure 3B), extended survival times (median OS had not been reached; Figure 3C), and wild-type or SDH mutations (80% wild-type, 13% SDH mutations; Figure 3D). Patients in the oldest age-group had characteristics more closely associated with adult-type GIST: slight male predominance (54% male), mixed primary tumor locations (37% stomach), shorter overall survival (median OS = 11.0 years), and dominated by KIT mutations (83%). Patients diagnosed between the ages of 18-35 appeared to be a mixture of pediatric and adult types, with all four characteristics (gender distribution, primary tumor location, mutational status and overall survival), falling between those expected of these previously described GIST types (Figure 3).

The median overall survival for all patients in the LRG registry was 11.7 years, (14.5 years for females and 10.3 years for males). Females predominated in pediatric-type GIST and survival is longer in pediatric-type GIST. This may partially explain the longer survival times of females when the entire range of ages at diagnosis was considered; however, females still retained a significant, albeit smaller, survival advantage even when only patients > 35 years old were considered (median OS = 11.0 years, females vs. 10.3 years males; P- value = 0.0006, HR = 1.517). Approximately 24% of LRG registry patients reported metastatic disease at time of diagnosis. This compares to 11% from a population-based study from the Rhône Alpes region of France [34], 15% from a population-based study in Western Sweden [1], 18% from a registry of patients from the United States [35], and 30% from the GOLD reGISTry (Global) [36]. Patients that initially presented with primary disease only, and later reported a recurrence, displayed a median overall survival > 11 years.

The 276 patients diagnosed over the age of 18, and reporting metastatic GIST at the time of diagnosis, represent the group of LRG registry patients that is easily defined, and expected to be most similar to those in the phase II and III metastatic GIST trials with respect to overall survival. The median overall survival for this sub-group within the cohort was 6.4 years from the time of diagnosis, reflective of the negative impact of metastatic disease at diagnosis upon overall survival. This is however, a longer overall survival time than typically reported for adult patients with metastatic disease, warranting further investigation. Recently it was reported that overall survival was better in the SWOG SO33 trial for centers that treated more than 15 patients versus those that treated 15 or fewer patients (median OS, 57 months vs 49 months)[37]. An intriguing question is whether patients receiving treatment or consultations at specialty centers that see many more GIST patients have better long-term outcomes than patients receiving care only at community- based practices. Another possibility is that approval of sunitinib in 2006 and off-label treatments such as nilotinib and sorafenib, which are commonly used by members (especially from the United States) with advanced disease, might be impacting overall survival compared to early trial results for metastatic disease.

The rate of mutational testing within this registry was 26.7% (30.5% for living members). While this is lower than typical research studies, it is much higher than the 6% testing rate reported in the reGISTry database of 822 patients treated in the United States [34]. The authors therein noted that the low mutational testing rate observed could be ascribed to the difference between actual clinical practice and guideline recommendations of the National Comprehensive Cancer Network (NCCN) and European Society of Medical Oncology (ESMO). They also noted that "Differences in response rates among reGISTry patients may also be attributed to differences in dosing patterns and treatment compliance in the 'real-world' versus clinical trial setting". The LRG registry represents a self- reported and international community, where mutational testing was carried out at a number of different centers. The level of testing can vary by center. For example, in the past, some centers tested for KIT mutations only, whereas testing for any SDH mutation is relatively rare; The only center within the United State of which we are aware that routinely screens for SDH mutations is the National Institutes of Health (NIH). The level of screening for SDHA mutations, which have only recently been reported, is unknown. As reliable immunohistochemical tools for detecting SDH deficiencies are identified, and become more wide-spread, we would expect the reported number of cases of both pediatric-type GIST and those with SDH mutations to increase.

Although imatinib appears to be less effective in pediatric- than adult-type GIST, relatively long survival times have been reported for patients with Carney's Triad and pediatric GIST [21]. These longer reported survival times pre-date the imatinib era and seem to be related to the relative indolence of the disease rather than any impact of imatinib or other targeted therapies. The LRG registry data confirms these observations for patients diagnosed below the age of 18. The median overall survival for these patients has not been reached and is significantly higher than older patients. In contrast, patients diagnosed over the age of 35 have shorter survival times in spite of effective drug therapies. Wild-type GIST/SDH-deficient patients had longer survival times, possible reflecting a high percentage of pediatric-type GIST in this group.

In our cohort, the ratio of females to males varied by the age at diagnosis. In the group of patients diagnosed below the age of 18, 75% (21/28) were female. The ratio of females to males remained higher until approximately age 35 and then dropped precipitously, and inverted with a slight male predominance in patients diagnosed after age 35. Adult- and pediatric-type GIST have different causes and respond differently to treatments. Pediatric GIST appears to be less dependent on KIT or PDGFRα than adult GIST, and may be somewhat dependent on IGF-1R signaling [38], as well as having deficiencies in the tumor suppressor activity of succinate dehydrogenase.

Since 2008, the National Institutes of Health, in collaboration with the Consortium for Pediatric and Wild-type GIST Research (CPGR), have been conducting biannual clinics for patients with wild-type and pediatric GIST. In addition to providing a resource for these patients, this group is collecting valuable data on these GIST subtypes. Given the rarity of pediatric GIST, it is important that patients and physicians are aware of this clinic. Our data suggests that, in addition to pediatric and adolescent patients, a significant portion of diagnosed "young adults" may also have the pediatric form of GIST. Pediatric-type GIST in adults was also recently reported by Rege and colleages in 16 patients (13 women, 3 men), with a median age at diagnosis of 31.5 years (range 19-56) [39].

Mutation testing is important for GIST patients including those diagnosed below the age of 35. Patients with characteristics associated with pediatric-type GIST (female gender, wild-type or SDH mutations, stomach primary tumors and epitheloid histology), should be referred to the NIH Pediatric and Wild-type GIST Clinic whenever possible. Immunohistochemical staining can also aid in identifying pediatric-type GIST, with a SDHB-deficiencies being strongly correlated with pediatric-type GIST [26,33]. All GIST patients diagnosed below the age of 18 should be referred to the clinic unless they have documented KIT or PDGFRα mutations. In addition, patients of any age with wild-type GIST may be eligible to participate in the clinic.

### Limitations

In this study we report on the survival of a large heterogeneous group of GIST patients of all ages that includes both metastatic and primary tumors. While patients in the LRG registry comprise a diverse group, there are differences when compared to the entire GIST population worldwide. LRG members are self-referred and participation is via the internet which could be prone to inclusion biases. For instance, younger patients are more likely to be internet-savvy than older patients, and patients in less developed countries may be excluded due to a lack of internet access, and other socioeconomic and language barriers. In addition, patients with advanced disease may be more motivated to participate than patients with less advanced disease. Being by and large self-referred, patients in the LRG registry may be more proactive, more likely to seek treatment at GIST referral centers, and more likely to register for, and potentially participate in, controlled clinical trials. In addition, a higher percentage of LRG patients have mutational testing (30.6% of living patients) compared to either those treated by academic centers (12%) or at community-based practices (1%) in the United States [35].

Patient-reported primary tumor locations should be interpreted with caution. In some cases, primary tumors may be adjacent, or adhered to other organs making reporting of a single, unique site difficult. For example, a doctor may report that the (primary) tumor was attached to the stomach, small intestine and pancreas. In most cases, operative notes and similar reports were not reviewed and in some cases primary tumor location cannot be conclusively determined even when these documents have been made available.

## Conclusions

Pediatric- and adult-type GIST have been previously characterized in clinical settings and the results of this long-term observational study of a self-reporting registry of patients confirm a number of significant prognostic factors for each from a large, diverse real-world cohort. Additionally, the convergence of these findings suggest that extra diligence be taken with "young adults" as pediatric-type GIST may present well beyond adolescence, particularly as these distinct sub-types have different causes, and consequently respond differently to treatments.

## Abbreviations

CI: Confidence interval; GI: Gastrointestinal; GIST: Gastrointestinal stromal tumor; HPF: High-power field; HR: Hazard ratio; KIT: c-KIT, CD117: v-kit Hardy-Zuckerman 4 feline sarcoma viral oncogene homolog; LRG: Life Raft Group; mets: Report of metastatic or advanced disease; NIH: National Institutes of Health; N/A: Not available; NBR, Not been reached; OS: Overall survival; PDGFRα: Platelet-derived growth factor receptor, alpha; SDH: Succinate dehydrogenase; wt: Wild-type.

## Competing interests

The authors declare that they have no competing interests.

## Authors' contributions

JC and NS conceived of the study. JC compiled and coded the data with respect to confidence in metastatic reports. JC and CDW analyzed the data and drafted the manuscript. JE was responsible for statistical analyses to which JC and CDW provided support. All authors read and approved the final manuscript.

## Pre-publication history

The pre-publication history for this paper can be accessed here:

http://www.biomedcentral.com/1471-2407/12/90/prepub
